# Ácidos Graxos Saturados e Implicações em Doenças Cardiovasculares

**DOI:** 10.36660/abc.20230612

**Published:** 2023-10-26

**Authors:** Matheus Augusto Callegari, Ricardo Luiz Damatto, Priscila Portugal dos Santos

**Affiliations:** 1 Universidade Estadual Paulista Júlio de Mesquita Filho Botucatu SP Brasil Universidade Estadual Paulista Júlio de Mesquita Filho Campus de Botucatu - Faculdade de Medicina de Botucatu, Botucatu, SP – Brasil; 2 Escola Universitária de Botucatu e Região Botucatu SP Brasil Escola Universitária de Botucatu e Região, Botucatu, SP – Brasil; 3 Faculdade de Ciências Sociais e Agrárias de Itapeva Itapeva SP Brasil Faculdade de Ciências Sociais e Agrárias de Itapeva, Itapeva, SP – Brasil

**Keywords:** Ácidos Graxos, Ácido Palmítico, Ácidos Esteáricos, Fatores de Risco Cardiovascular, Lipoproteínas

As doenças cardiovasculares (DCV) são a principal causa de mortalidade, sendo responsáveis por 17,9 milhões de mortes em 2019, 32% de todas as mortes globais.^1^ É importante detectar precocemente a DCV para iniciar o tratamento. Para isso, existem vários fatores de risco padrão na predição de DCV, mas o interesse em encontrar biomarcadores mais sensíveis e precisos é intenso. Gonçalinho et al.^2^ propõem que o uso de biomarcadores de inflamação e disfunção endotelial pode refletir DCV e predizer pior prognóstico, independentemente dos fatores de risco tradicionais. A maioria das DCV pode ser prevenida abordando fatores de risco comportamentais, como dieta. A ingestão de gordura na dieta é um dos principais fatores de risco modificáveis implicados na causa da DCV.^3^ Gonçalinho et al.^2^ mostraram que o ácido esteárico (AE) está associado a biomarcadores inflamatórios e disfunção endotelial em indivíduos com risco cardiovascular.

Para estudar a associação dos ácidos graxos (AG) com fatores de risco cardiovascular, Gonçalinho et al.^2^ usaram AG de fosfolipídios de membrana de glóbulos vermelhos (GV) como um biomarcador do status de AG. Esta análise tem sido utilizada, uma vez que estes AGs podem ser incorporados a partir das lipoproteínas séricas.^4^ Além disso, oferece uma vantagem sobre a análise no plasma, porque os GV duram em média 120 dias, em comparação com 3 semanas para os lípidos plasmáticos, refletindo uma melhor ingestão de AG na dieta a longo prazo.^5^ Porém, é importante destacar que o custo de preparação de hemácias supera o método plasmático e que existem diferentes estratégias analíticas.^6 , 7^ Portanto, para que o AG dos fosfolipídios da membrana das hemácias se torne um biomarcador do estado nutricional do AG dietético, na prática clínica, continua sendo necessário uma investigação sobre custo-efetividade e padronização de métodos.^8^

Outra análise interessante é o AG intracelular das hemácias. Embora os GV não possuam mitocôndrias para oxidar o AG, estudos mostraram que o AG plasmático pode ser incorporado aos triacilglicerois (TGs) dos GV. Esses TGs são enriquecidos em ácidos graxos saturados (AGS) e empobrecido em ácidos graxos insaturados, contrastando com o perfil altamente insaturado dos fosfolipídios da membrana. Portanto, um dos dois métodos pode ser mais preciso dependendo do AG de interesse.^4^

A influência dos AGS no risco cardiovascular ainda é controversa, como mostraram Gonçalinho et al.,^2^ que não observaram associação entre ácido palmítico (AP) e fatores de risco cardiovascular, embora essa associação seja conhecida na literatura. Contudo, mostraram que o AE está associado a biomarcadores de risco cardiovascular, enquanto outros autores observaram um efeito neutro ou benéfico do AE.^9^ Essas diferenças podem ser devido à posição do AG na molécula de TG (sn-1, sn-2 e sn-3), uma vez que a posição determina as características bioquímicas e físicas da gordura e pode influenciar sua absorção no intestino, metabolismo nos enterócitos, subsequente metabolismo dos quilomícrons e distribuição nos tecidos. Isto pode levar a diferentes efeitos sobre os lipídios plasmáticos e risco cardiovascular. Gorduras contendo AP e AE na posição sn-2 são melhor digeridas e consideradas mais aterogênicas.^12^

De fato, estudos mostraram que o óleo de palma, que tem aproximadamente <10% do total de AP na posição intermediária, não aumenta as concentrações de colesterol.^12 , 13^ Além disso, um estudo com manteiga de cacau (rica em AE na posição 1,3) mostrou um efeito neutro do AE na concentração de colesterol.^12 , 14^ Assim, é importante enfatizar que os efeitos dos AGS na saúde dependem não apenas do AG específico, mas também das fontes, quantidade e qualidade do alimento, bem como do grau de processamento e do efeito sinérgico dos demais compostos. Portanto, estudos recentes aconselham que o preconceito contra os alimentos ricos em AGS deve ser substituído com vista a recomendar dietas constituídas por alimentos saudáveis.^9 - 11^

Outro resultado importante apresentado por Gonçalinho et al.^2^ foi a associação do AE com melhora do perfil lipídico, mesmo quando associado a biomarcadores de risco cardiovascular. Estudos levantaram a hipótese de que não a concentração de lipoproteínas, mas a funcionalidade das partículas de HDL e a qualidade das partículas de LDL são mais importantes para prever o risco cardiovascular.^9 , 15^ Portanto, seria interessante que novos estudos abordassem essas novas análises.
