# Supplementary material for: Ácidos Graxos Saturados e Implicações em Doenças Cardiovasculares
Source: Arq Bras Cardiol. 2023 Oct 26;120(8):e20230612. [Article in Portuguese] doi: 10.36660/abc.20230612 (PMC12080653; doi:10.36660/abc.20230612)
Supplement: Translation

# Translation: Saturated Fatty Acids and Implications for Cardiovascular Disease

**Authors:** Matheus Augusto Callegari, Ricardo Luiz Damatto, Priscila Portugal dos Santos

**Affiliations:** 1 Brasil Universidade Estadual Paulista Júlio de Mesquita Filho Campus de Botucatu - Faculdade de Medicina de Botucatu, Botucatu, SP – Brasil; 2 Brasil Escola Universitária de Botucatu e Região, Botucatu, SP – Brasil; 3 Brasil Faculdade de Ciências Sociais e Agrárias de Itapeva, Itapeva, SP – Brasil

**Keywords:** Fatty Acids, Palmitic Acid, Stearic Acids, Heart Disease Risk Factors, Lipoproteins

Cardiovascular diseases (CVD) are the leading cause of mortality, accounting for 17.9 million deaths in 2019, 32% of all global deaths.^1^It is important to detect CVD early to start treatment. For this, there are several standard risk factors in CVD prediction, but interest in finding more sensitive and accurate biomarkers is intense. Gonçalinho et al.^2^propose that the use of inflammation and endothelial dysfunction biomarkers may reflect CVD and predict worse prognosis, regardless of traditional risk factors. Most CVDs can be prevented by addressing behavioral risk factors like diet. Dietary fat intake is one of the major modifiable risk factors implicated in the causation of CVD.^3^Gonçalinho et al.^2^showed that stearic acid (SA) is associated with inflammatory biomarkers and endothelial dysfunction in individuals at cardiovascular risk.

To study the association of fatty acids (FA) with cardiovascular risk factors, Gonçalinho et al.^2^used FA from membrane phospholipids of red blood cells (RBC) as a biomarker of FA status. This analysis has been used since these FAs can be incorporated from the serum lipoproteins.^4^Additionally, it offers an advantage over analysis of plasma because RBC last on average 120 days compared to 3 weeks for plasma lipids, reflecting better long-term dietary FA intake.^5^However, it is important to highlight that the cost of preparing RBC exceeds the plasmatic method and that there are different analytical strategies.^6 , 7^Therefore, for FA from RBC membrane phospholipids to become a biomarker of the nutritional status of dietary FA in clinical practice, there remains a need for investigations of cost-effectiveness and method standardization.^8^

Another interesting analysis is the intracellular FA of RBC. Although RBCs lack mitochondria to oxidize FA, studies showed that plasma FA can be incorporated into RBC triacylglycerols (TG). These TG are enriched in saturated FA (SFA) and depleted in unsaturated FA, contrasting the highly unsaturated profile in membrane phospholipids. Therefore, one of the methods may be more accurate depending on the FA of interest.^4^

The influence of SFA on cardiovascular risk is still controversial, as shown by Gonçalinho et al.,^2^who did not observe an association between palmitic acid (PA) and cardiovascular risk factors, although this association is known in the literature. However, they showed that SA is associated with biomarkers of cardiovascular risk, while other authors observed a neutral or beneficial effect of AS.^9 - 11^These differences may be due to the position of FA in TG molecule (sn-1, sn-2, and sn-3) since it determines the biochemical and physical characteristics of the fat and may influence their absorption from the gut, metabolism in enterocytes, subsequent chylomicron metabolism and distribution into tissues. This can lead to different effects on plasma lipids and cardiovascular risk. Fats containing PA and SA in the sn-2 position are better digested and considered more atherogenic.^12^

In fact, studies showed that palm oil, which has approximately <10% of the PA total in the intermediate position, does not increase cholesterol concentrations.^12 , 13^Additionally, a study with cocoa butter (rich in SA at position 1,3) showed a neutral effect of SA on cholesterol concentration.^12 , 14^Thus, it is important to emphasize that the health effects of SFA depend not only on the specific FA but also on the sources, quantity and quality of the food, as well as the degree of processing and synergistic effect of the other compounds. Therefore, recent studies have advised that the bias against foods rich in SFA should be replaced with a view toward recommending diets consisting of healthy foods.^9 - 11^

Another important result shown by Gonçalinho et al.^2^was the association of SA with an improvement in the lipid profile, even when associated with cardiovascular risk biomarkers. Studies have hypothesized that not the concentration of lipoproteins but the functionality of HDL particles and the quality of LDL particles are more important for predicting cardiovascular risk.^9 , 15^Therefore, it would be interesting for further studies to address these new analyses.
